# Supplementary material for: Identification of photoperiod-induced specific miRNAs in the adrenal glands of Sunite sheep (Ovis aries)
Source: Front Vet Sci. 2022 Jul 22;9:888207. doi: 10.3389/fvets.2022.888207 (PMC9354845; doi:10.3389/fvets.2022.888207)
Supplement: Supplementary file 1 [file Data_Sheet_1.ZIP › Supplementary file/miRDeep2 file.zip!/miRDeep2 file/Novel_Structure/SPLP42A2/Novel_478.pdf]

Provisional ID	:	NC_019461.2_12820
Score total	:	1.8
Score for star read(s)	:	-1.3
Score for read counts	:	0
Score for mfe	:	1.5
Score for randfold	:	1.6
Score for cons. seed	:	
Total read count	:	13
Mature read count	:	10
Loop read count	:	0
Star read count	:	3

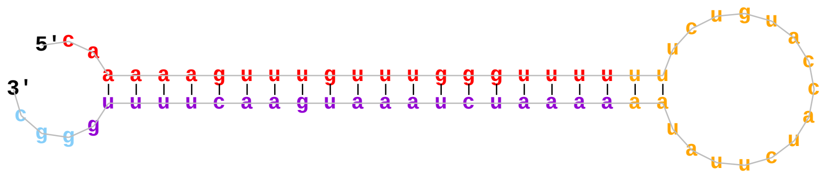

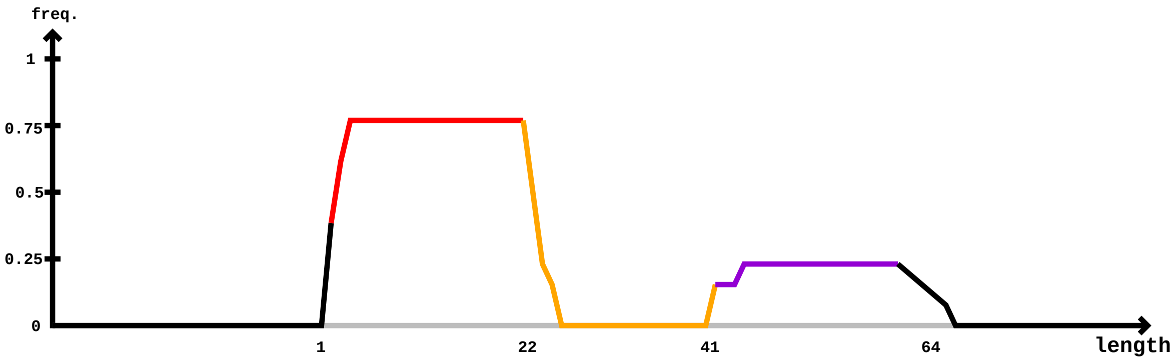

Mature **Star**

```
5'- ccaaggagaauuuaauauuggauuggccaaaaaguuuguuuggguuuuuuucuguaccaucuuauaaaaaaucuaaaugaacuuuugggccaacccaauagugagaagaacc   -3'    obs
    ccaaggagaauuuaauauuggauuggccaaaaaguuuguuuggguuuuuuucuguaccaucuuauaaaaaaucuaaaugaacuuuugggccaacccaauagugagaagaacc          exp
    ...........(((.((((((.((((((.(((((((((((((((((((((...............))))))))))))))))))))).)))))).)))))).)))........    reads     mm      sample
    ...........................caaaaaguuuguuuggguuuu................................................................    3         0       seq
    ...........................caaaaaguuuguuuggguuuuu...............................................................    2         0       seq
    ............................aaaaaguuuguuuggguuuuuu..............................................................    1         0       seq
    ............................aaaaaguuuguuuggguuuuuCu.............................................................    2         1       seq
    .............................aaaaguuugCuuggguuuu................................................................    1         1       seq
    .............................aaaaguuuguuuggguuuuu...............................................................    1         0       seq
    ...................................................................aaaaucuaaaugaacuuuuU.........................    2         1       seq
    ......................................................................aucuaaaugaacuuuugggcca....................    1         0       seq
```
